# A Comparative Study of Divergent Surface Acoustic Wave Beams’ Generation on an Y128° Lithium Niobate Using Various Types of Interdigital Transducers

**DOI:** 10.3390/s25041067

**Published:** 2025-02-11

**Authors:** Marc Duquennoy, Dame Fall, Nada Ben Jafela, Nikolay Smagin, Zakariae Oumekloul, Lynda Chehami, Emmanuel Moulin, Mohammadi Ouaftouh

**Affiliations:** IEMN (UMR CNRS 8520), University Polytechnique Hauts-de-France, CNRS, University Lille, INSA HdF, F-59313 Valenciennes, France; dame.fall@uphf.fr (D.F.); nada.benjafela@uphf.fr (N.B.J.); nikolay.smagin@uphf.fr (N.S.); zakariae.oumekloul@uphf.fr (Z.O.); lynda.chehami@uphf.fr (L.C.); emmanuel.moulin@uphf.fr (E.M.); mohammadi.ouaftouh@uphf.fr (M.O.)

**Keywords:** interdigital transducers, SAW sensor, surface acoustic waves, SAW divergence, focused IDT, laser Doppler vibrometry

## Abstract

This study focuses on the possibility of generating divergent surface acoustic waves (SAWs) with interdigital transducers (IDTs) deposited on Y128° Lithium Niobate for non-destructive testing applications, particularly in the context of manufacturing layer-on-substrate systems for microelectronic components. The selected approach is to diffuse the SAWs over a large surface area and in various directions in order to analyze the structure and detect any defects when using the well-known passive imaging by correlating the diffuse acoustic field. The introduction of SAWs is achieved using offset interdigital transducers that make acoustic contact with the sample under analysis without causing damage. The considered IDTs are characterized based on criteria for maximizing the divergence angle, maximizing SAW emission amplitude, and minimizing the acoustic contact area. Three IDT configurations were tested to compare their performance: (i) a transducer with circular electrodes emitting from its wide convex end; (ii) a circular IDT emitting from its narrow concave end; and (ii) a narrow transducer with an aperture close to two wavelengths. It was shown that the second configuration provides the highest SAW amplitude, which is important while measuring the diffuse acoustic field. Nevertheless, the third one was particularly efficient in terms of reducing the contact area.

## 1. Introduction

This study investigates the possibility of obtaining divergent surface acoustic waves (SAWs) on the surface of any material, particularly on silicon wafers. The context of this study addresses the need to generate SAWs over a wide area and in various directions to facilitate SAW diffusion for passive non-destructive testing. The reverberation of acoustic waves allows for a diffuse field to be obtained, Ref. [1], ultimately producing a coda [2] (a high number of multiple reflections) that can be used to characterize materials, perform non-destructive structural testing, and identify potential defects [3,4,5,6]. The use of a coda wave for NDT/E has been previously investigated for corrosion detection [7], defect localization [8] in elastic plates [9,10], or biofilm monitoring [11,12]. In order to apply this technique for layer-on-substrate characterization, a suitable type of transducer has to be designed and realized.

Among the various methods used to generate Rayleigh-type [13] SAWs, offset interdigital transducers (IDTs) were selected for this study because they offer several advantages. First, they allow for SAWs to be generated on any sample, even transparent ones, without the risk of damage. No sample preparation is necessary. Moreover, this type of transducer allows for the frequency range in which SAWs are generated to be selected [14,15].

The originality of this work focuses on the technological part for SAW-IDT conception capable of generating high-frequency noise (dozens of MHz). The objective is to control and image micro-scale defects in silicon wafers using the reverberation of surface acoustic waves [16,17]. The latter is outside of the scope of this paper and will be the subject of another publication.

A key challenge was the need to minimize the contact area between the IDT and the sample where SAW reverberation is desired. This contact limitation between the transducer and the silicon wafer is important because, while necessary for transferring SAWs from the IDT to the structure to be characterized, this contact can absorb part of the reverberated acoustic energy, which is unsuitable for the generation of long duration coda signals. A large number of reverberations is required to effectively use a coda and achieve non-destructive characterization through reverberations [18]. Therefore, minimizing the contact area between the transducer and the silicon wafer is imperative.

In this study, several IDTs were developed and manufactured to compare their performance in meeting the above requirement. Three configurations were tested with IDTs operating at a frequency of 15 MHz to compare the potential of each configuration. The piezoelectric substrate used is Y128° LiNbO_3_, chosen for its high electromechanical coupling coefficient (K^2^ = 5.4%), ensuring the efficient conversion of electrical energy into high-frequency mechanical vibrations.

## 2. Excitation of Divergent SAW Beams with Various Types of IDTs

**Figure 1:**
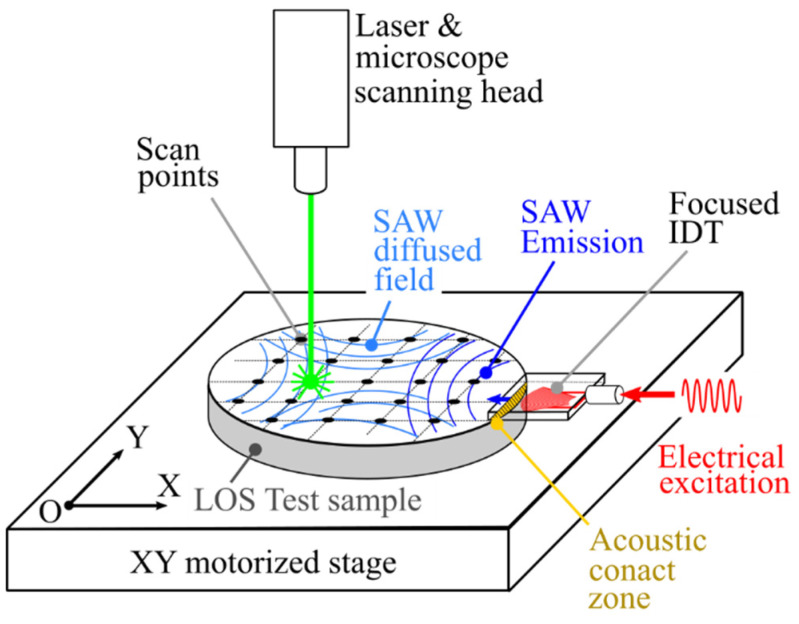
Schematic diagram of SAW introduction on an LOS sample using an offset IDT and SAW displacement recording with a laser vibrometer.

To introduce SAWs to coated silicon wafers at a frequency of 15 MHz, it becomes difficult to use wedge sensors, commonly employed in non-destructive testing (NDT). These sensors are generally limited to 10 MHz and cannot produce a divergent acoustic beam. Furthermore, laser ultrasound [19] techniques are challenging to use because they can damage coatings deposited on wafers during component fabrication. Given these conditions, SAW excitation via IDTs was preferred [20,21,22]. This method has already proven to be effective and offers numerous possibilities, such as selecting frequencies up to several hundred MHz and adjusting divergence by modifying the curvature of the electrodes [23,24,25]. Additionally, the IDTs are offset relative to the structures being characterized to excite the SAWs. In these conditions, there are no restrictions on the nature of the coatings or any alteration of the surfaces being characterized (Figure 1).

**Figure 2:**
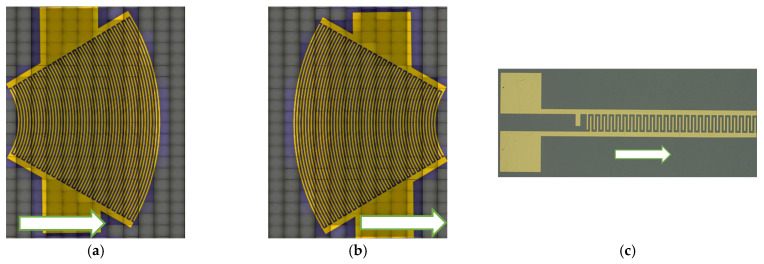
Three configurations of IDTs used to generate divergent SAW beams on LiNbO_3_ substrate using LDV: (**a**) divergent IDT; (**b**) convergent IDT; (**c**) narrow IDT. The SAW emission in a left-to-right direction.

Three configurations of IDTs were considered for generating divergent acoustic beams: (i) a transducer with circular electrodes emitting from its wide, convex end (Figure 2a); (ii) the same focused IDT with circular electrodes, but this time emitting from its narrow, concave end (Figure 2b); and (iii) a rectilinear transducer emitting through a narrow aperture equal to 1.87 wavelengths (Figure 2c). Thus, the SAW beam divergence is naturally obtained in the first configuration with a convex circular aperture. The second configuration uses a concave aperture to initially produce a converging beam, which becomes divergent after the focal point. The third configuration achieves divergence through a rectilinear IDT with a narrow aperture. For ease of reading, we will refer to these configurations as the “divergent IDT”, the “convergent IDT”, and the “narrow IDT”.

In this study, the acoustic field profiles were recorded directly on the transducers’ LiNbO_3_ substrate to compare their performance and identify the optimal configuration based on the criteria of maximizing the divergence angle, maximizing the SAW emission amplitude, and minimizing the acoustic contact area. The transducers were fabricated using optical lithography, which offers a resolution of approximately 1 µm. To ensure consistency, the width and spacing between the electrodes were systematically rounded to the nearest micrometer. The devices were manufactured through a lift-off process performed at the IEMN technological center. This method enabled the high-resolution deposition of gold electrodes onto niobate wafers, ensuring precise and reliable fabrication of the SAW devices [21].

## 3. Experimental Setup for Measuring Displacement Fields

**Figure 3:**
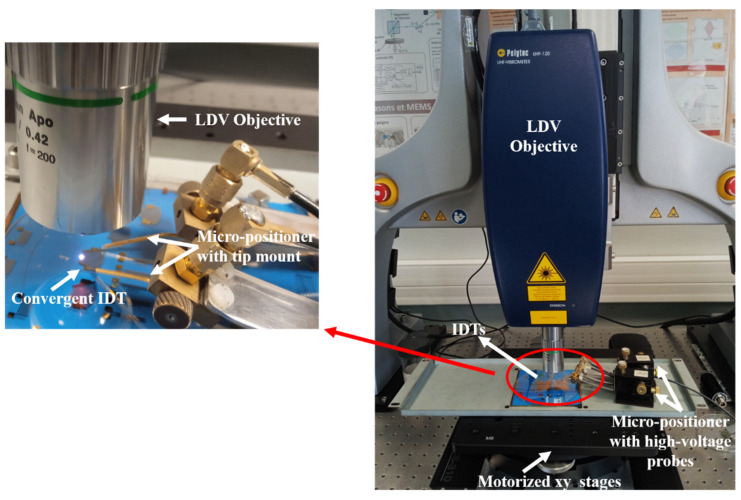
The measurement system consisting of the Polytec UHF-120 LDV, displacement table, transducer, and micro-positioner with probes.

Displacement measurements to determine acoustic fields were performed on a platform called WAVESURF based on a laser Doppler vibrometer (LDV) (Figure 3) [26,27,28]. The LDV works on the principle of optical interference, measuring the phase shift in the reflected laser beam caused by displacement normal to the sample surface. Measurements are taken directly on the transducer to map the SAW acoustic field profiles. The transducers are excited using high-voltage probes connected to a micro-positioner for maintaining the probes.

**Figure 4:**
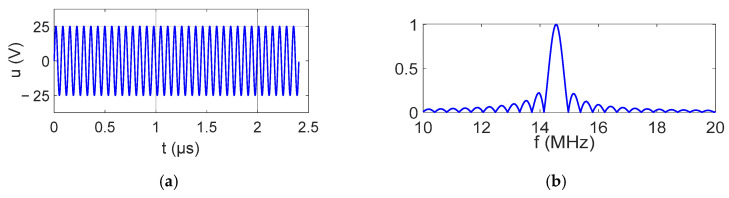
Electrical signal imposed on the IDT: (**a**) time signal; (**b**) signal spectrum.

The IDT was excited using a waveform generator connected to a 50 W power amplifier. The electrical signal applied to the IDT was a tone burst of 35 sinusoidal cycles with a 50 Vpp amplitude. The signal and its bandwidth are shown in Figure 4.

## 4. Results for the Divergent IDT

**Figure 5:**
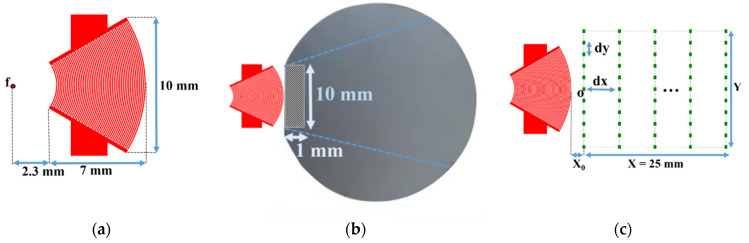
(**a**) Schematic of the divergent IDT; (**b**) contact area (hatched zone) between the divergent IDT and the silicon wafer; and (**c**) scanning points for the displacement field measurement on the LiNbO_3_ substrate.

First, a divergent IDT was tested, where the electrodes were shaped as circular arcs with a common center of curvature. This solution is the most natural choice for achieving effective wave divergence. The IDT was constructed with 25 pairs (Table 1) of electrodes deposited on a Y128° lithium niobate substrate [29]. The electrode widths were approximately 65 µm, corresponding to a quarter wavelength at 15 MHz, given the SAW propagation speed in lithium niobate at around 4000 m/s. The angular aperture was ±30 degrees, with an inner curvature radius of 2.8 mm and an outer curvature radius of 9.3 mm for the annular sector formed by the IDT. The width at the outer edge of the IDT is 10 mm, and the focal point is located 2.3 mm from the inner edge of the IDT (Figure 5a). This configuration requires a contact area of about 1 × 10 mm (10 mm^2^) between the SAW emitter (lithium niobate) and the silicon wafer coating to be characterized (Figure 5b).

Scanning points for the SAW displacement field measurement on the LiNbO_3_ substrate are shown in Figure 5c. Twenty-five scanning lines parallel to the IDT electrodes were defined. The length Y of each line was 1.5 times the maximum length of the IDT (15 mm), with lines spaced by d*x* = 1 mm. Each line contained *N*_y_ = 51 scan points with a step size d*y* = 0.3 mm. The distance between the last electrode and the first scan line was defined as *x*_0_ = 0.5 mm. The displacement field on the lithium niobate transducer was thus recorded over an area of 25 × 15 mm^2^ and is shown in Figure 5c.

**Figure 6:**
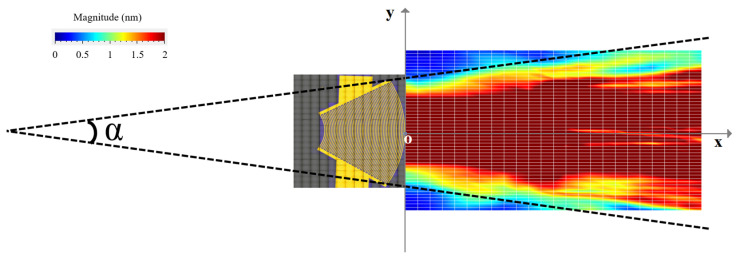
Displacement field obtained for the divergent IDT with LDV.

The displacement field obtained for the divergent IDT is shown in Figure 6. The displacements induced by the SAWs are of the order of 10 s of nanometers, and the acoustic beam diverges with an angle α around 15 degrees. The exact divergence angle at −3 dB and −10 dB will be presented in the discussion section. To better illustrate the divergence, color saturation was applied to displacement levels equal to or above 10% of the maximum displacement.

**Figure 7:**
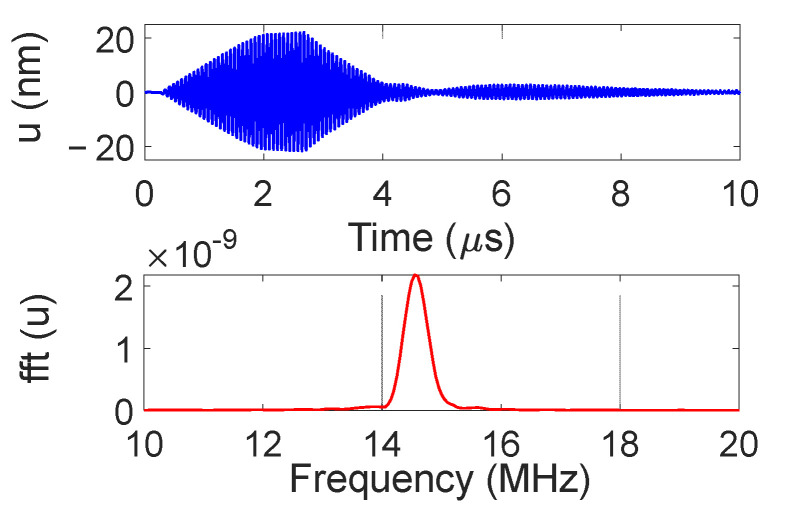
Temporal and frequency response of the normal displacement measured at point “o” for the divergent IDT.

**Figure 8:**
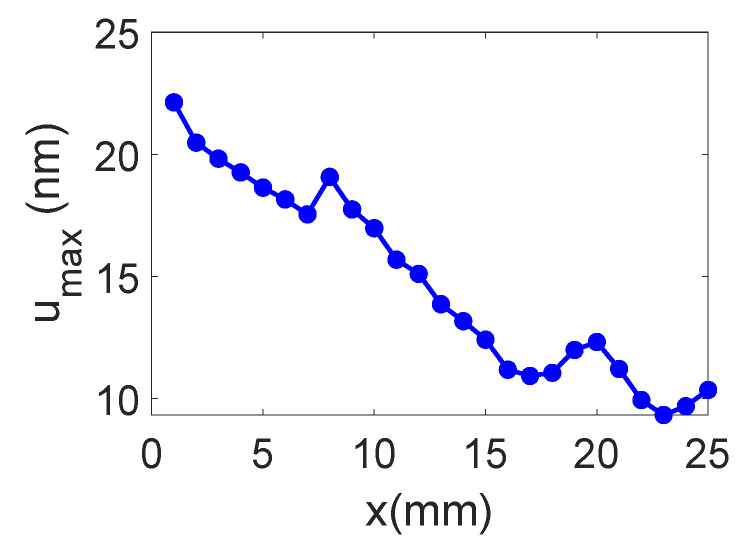
Maximum normal displacement for each profile obtained with the divergent IDT.

The temporal and frequency response of the normal displacement measured at point “o” for the divergent IDT is shown in Figure 7. The displacement amplitude of approximately ±20 nm is satisfactory for the NDT goals, and the signal-to-noise ratio is quite acceptable (37 dB). Figure 8 presents the maximum normal displacement for each profile (for a given *x*-coordinate) obtained with the divergent IDT. The decay in displacement is due to the attenuation of surface acoustic waves on the lithium niobate and the beam divergence, which reduces power density.

**Figure 9:**
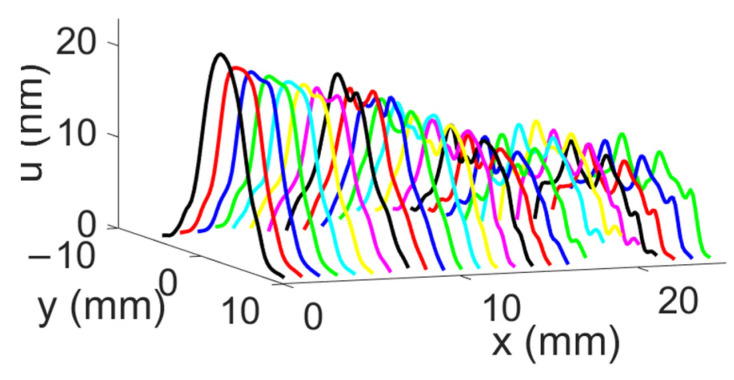
Maximum normal displacement for each profile obtained with the divergent IDT. Displacement field profiles obtained with the divergent IDT.

**Figure 10:**
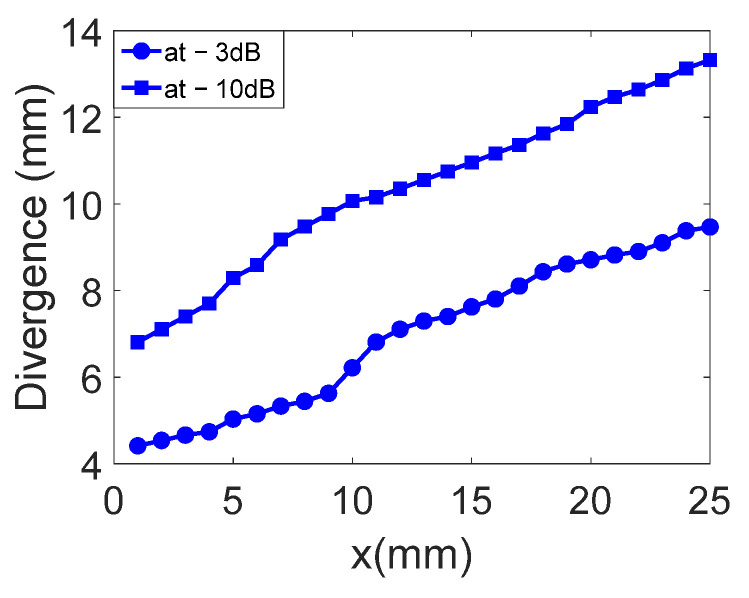
Beam width in millimeters at −3 dB and −10 dB is estimated for each profile obtained with the divergent IDT.

The displacement field profiles obtained for the divergent IDT are shown in Figure 9 (The colors in Figure 9 are used for distinguishing between different displacement field profiles and do not represent any specific physical parameter). This representation complements Figure 6 and helps to verify the divergence of the acoustic beam. In Figure 10, the beam width in millimeters at −3 dB and −10 dB is estimated for each profile obtained with the divergent IDT.

## 5. Results for the Convergent IDT

**Figure 11:**
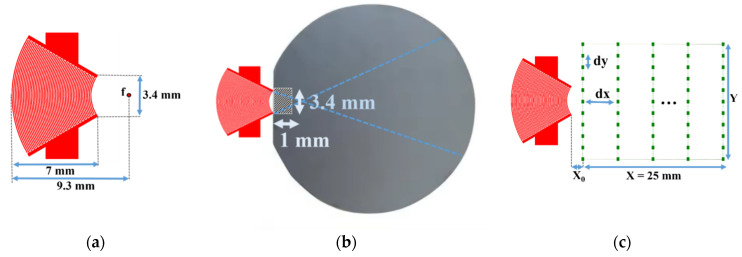
(**a**) Schematic of the convergent IDT; (**b**) contact area (hatched zone) between the convergent IDT and the silicon wafer; and (**c**) scanning points for the displacement field measurement on the LiNbO_3_ substrate.

Next, a concave aperture IDT was tested to achieve divergence. This IDT has the same characteristics as the previous one (25 pairs of electrodes deposited on a Y128°-cut lithium niobate substrate), as shown in Figure 11a and Table 1. This convergent IDT was designed with electrodes in the shape of circular arcs sharing the same center of curvature. This design initially focuses on the SAWs at a focal point, after which the waves become divergent. The width at the inner edge of the IDT is 3.4 mm.

**Figure 12:**
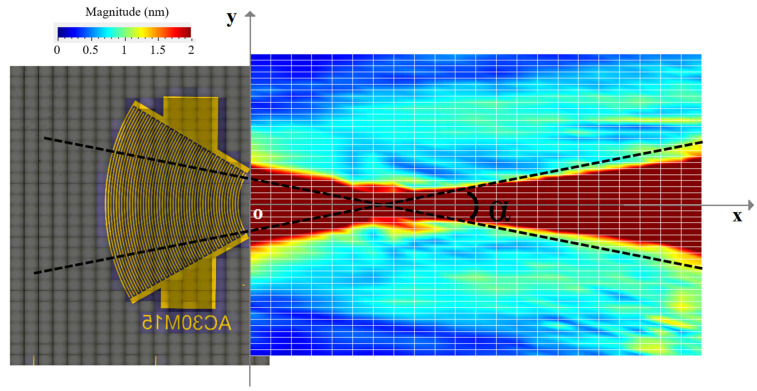
SAW displacement field obtained with the convergent IDT.

**Figure 13:**
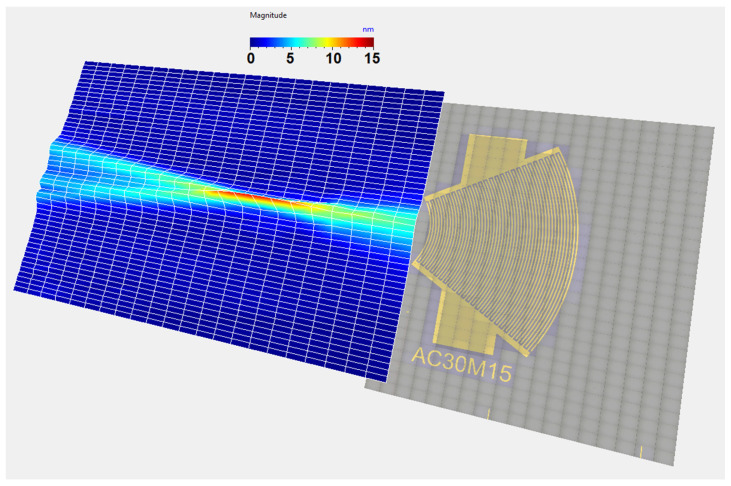
Three-dimensional representation of the SAW displacement field obtained with the convergent IDT.

This configuration requires a contact area of about 1 × 3.4 mm (3.4 mm^2^) between the IDT and the silicon wafer (Figure 11b). To verify the capability of the convergent IDT to produce a divergent beam, the acoustic field profile was measured with LDV using the same scanning settings as described above for the divergent IDT. The distance between the last electrode and the first scan line is defined by x0 = 0.5 mm (Figure 11c). The displacement field obtained with the convergent IDT is shown in 2D in Figure 12 and in 3D in Figure 13. The SAW-induced displacements are on the order of nanometers, and the acoustic beam diverges with an angle of approximately 15 degrees.

**Figure 14:**
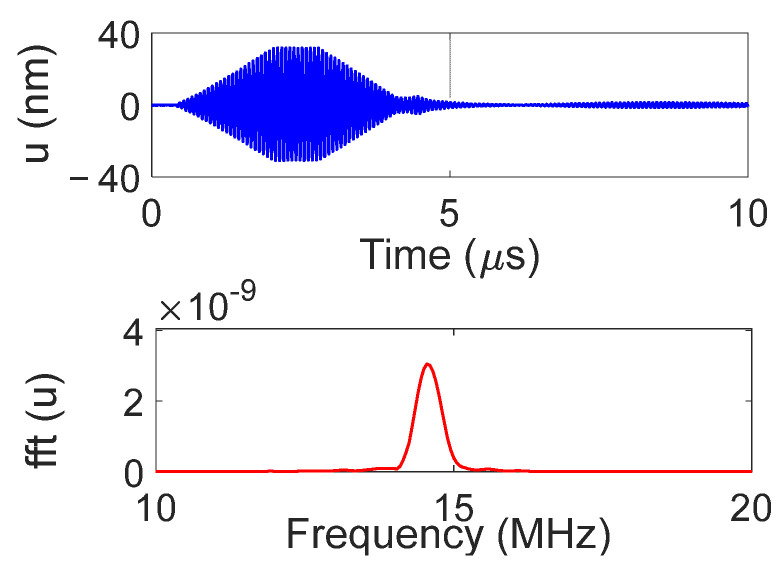
Temporal and frequency response of the normal displacement measured at point “o” for the convergent IDT.

**Figure 15:**
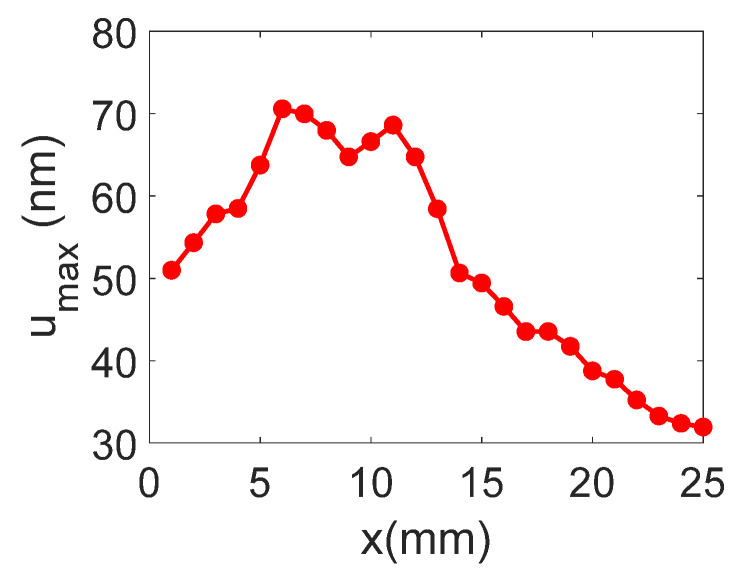
Maximum normal displacement for each scan line obtained with the convergent IDT.

The temporal and frequency response of the normal displacement measured at point “o” for the convergent IDT is shown in Figure 14. The displacement amplitude of approximately ±30 nm is satisfactory for the NDT goals, and the signal-to-noise ratio is decent (40 dB). Figure 15 presents the maximum normal displacement for each scan line obtained with the convergent IDT. The decay in displacement is again due to wave attenuation and beam divergence.

**Figure 16:**
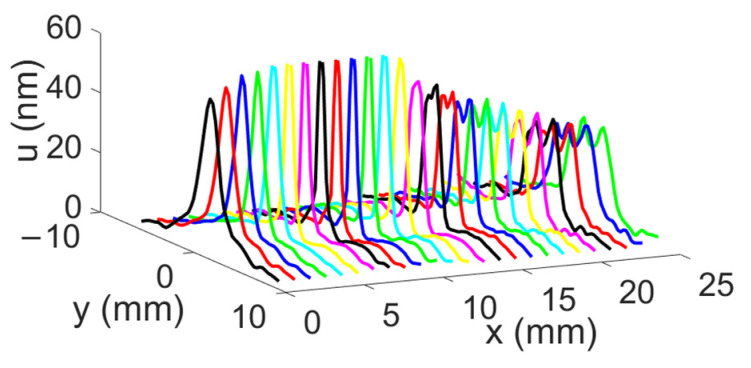
SAW displacement field profiles obtained with the convergent IDT.

**Figure 17:**
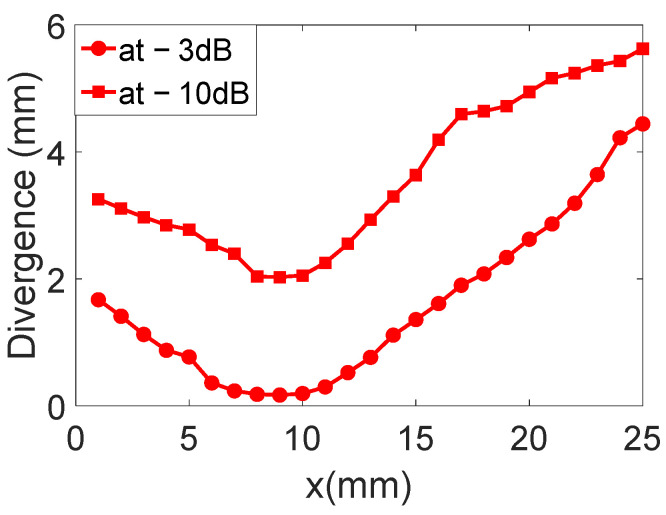
Beam widths at −3 dB and −10 dB for each profile obtained with the convergent IDT.

The displacement field profiles obtained for the convergent IDT are shown in Figure 16 (The colors in Figure 16 are used for distinguishing between different displacement field profiles and do not represent any specific physical parameter). This representation complements Figure 12 and verifies the beam’s divergence. Figure 17 estimates the beam width at −3 dB and −10 dB for each profile obtained with the convergent IDT.

## 6. Results for the Narrow IDT

**Figure 18:**
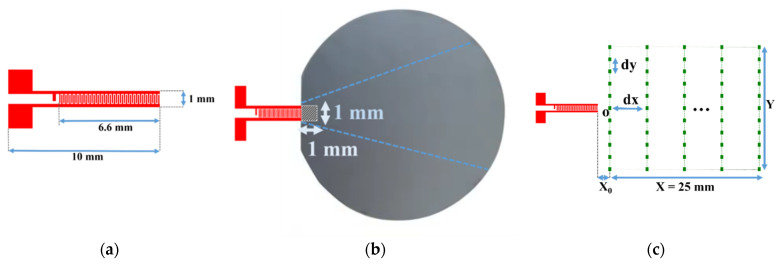
Narrow aperture-type IDT: (**a**) schematic of photolithography mask; (**b**) contact area (hatched zone) between the IDT and the silicon wafer; (**c**) scanning points for the displacement field measurement on the LiNbO_3_ substrate.

Finally, a narrow IDT was tested, where the electrodes were straight and narrow. This configuration reduces the IDT’s width to achieve divergence. The IDT was built with 25 pairs of electrodes with an aperture width of 1 mm (Table 1). The electrodes had widths of approximately 65 µm, corresponding to a quarter wavelength at 15 MHz, and the aperture was equal to 1.87 wavelengths (0.5 mm). This IDT is shown in Figure 18a.

**Figure 19:**
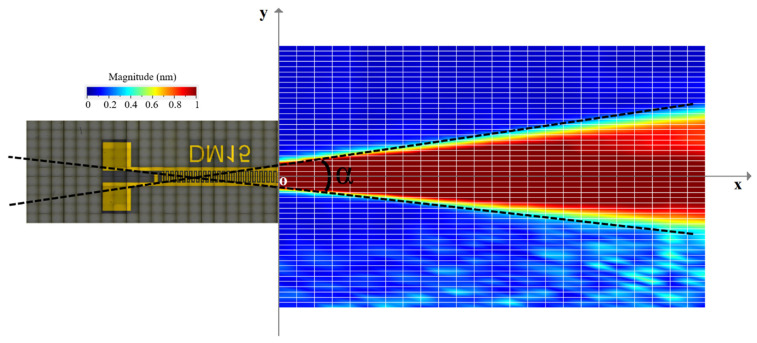
SAW displacement field obtained with the narrow IDT.

**Figure 20:**
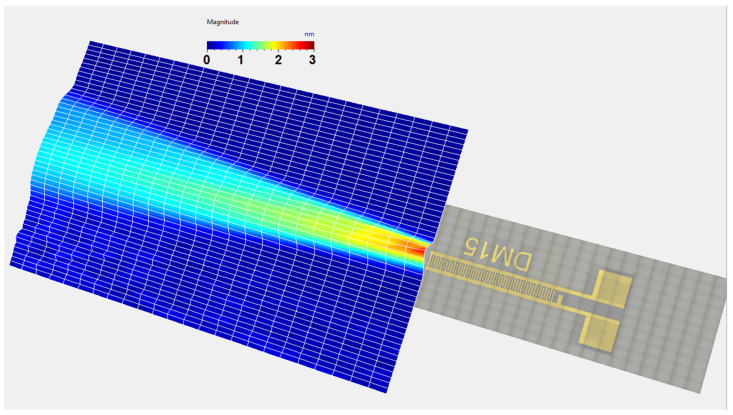
Three-dimensional representation of the SW displacement field obtained with the narrow IDT.

This configuration requires a contact area of about 1 × 1 mm (1 mm^2^) between the IDT and the silicon wafer (Figure 18b). The displacement field profile was measured in the same way as for the other IDTs (Figure 18c). The displacement field for the narrow IDT is shown in 2D in Figure 19 and in 3D in Figure 20. The SAW-induced displacements are on the order of nanometers, and the beam diverges with an angle of approximately 15 degrees.

**Figure 21:**
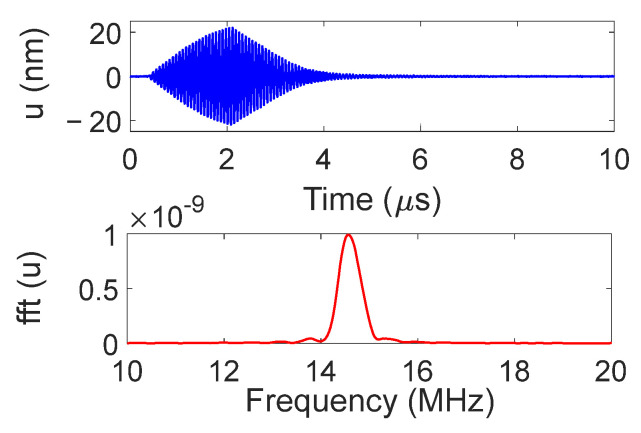
Temporal and frequency domain IDT response measured at point “o” for the narrow IDT.

**Figure 22:**
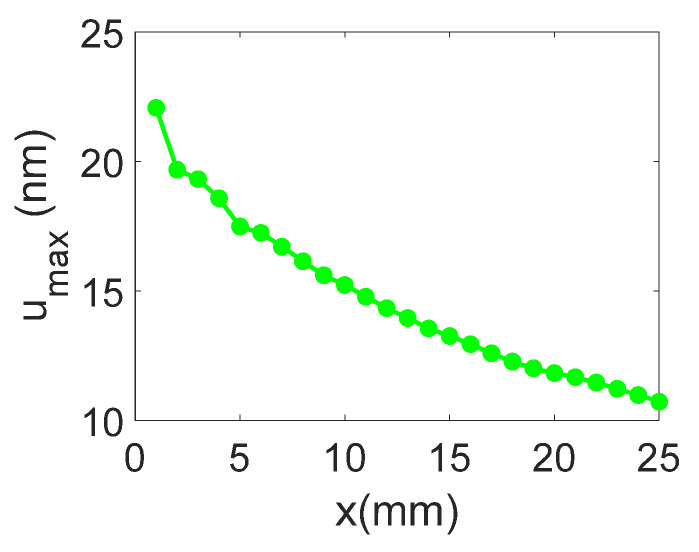
Maximum SAW displacement for each scan profile obtained with the narrow IDT.

The temporal and frequency response of the normal displacement measured at point “o” for the narrow IDT is shown in Figure 21. The displacement amplitude of approximately ±20 nm is satisfactory for the NDT purposes, while the signal-to-noise ratio is 37 dB. Figure 22 presents the maximum normal displacement for each scan line obtained with the narrow IDT.

**Figure 23:**
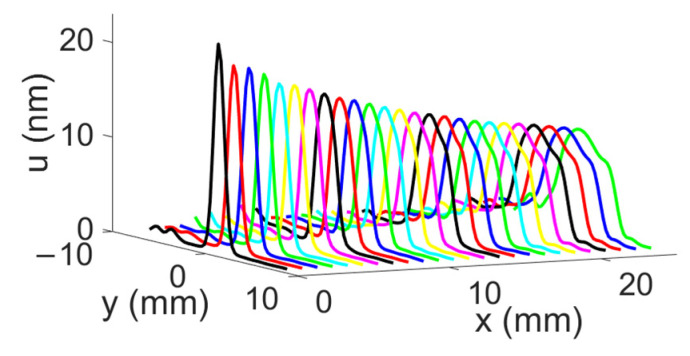
SAW displacement field profiles obtained with the narrow IDT.

**Figure 24:**
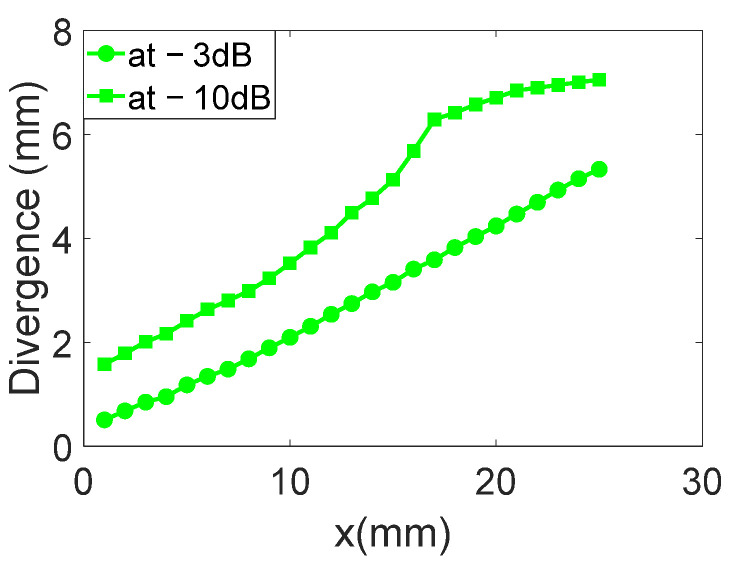
Beam widths at −3 dB and −10 dB for each profile of the narrow IDT.

The displacement field profiles obtained for the narrow IDT are shown in Figure 23 in addition to the abovementioned representation in Figure 20 (The colors in Figure 23 are used for distinguishing between different displacement field profiles and do not represent any specific physical parameter). Finally, Figure 24 estimates the beam width at −3 dB and −10 dB for each profile obtained with the narrow IDT.

## 7. Discussions

Regarding the need to minimize the contact area between the IDT and the sample for SAW reverberation, the three proposed solutions are significantly different, with contact areas of 10 mm^2^, 3.4 mm^2^, and 1 mm^2^, respectively. The third solution (narrow IDT) reduces the contact area by a factor of 10 compared to the first solution (divergent IDT).

**Figure 25:**
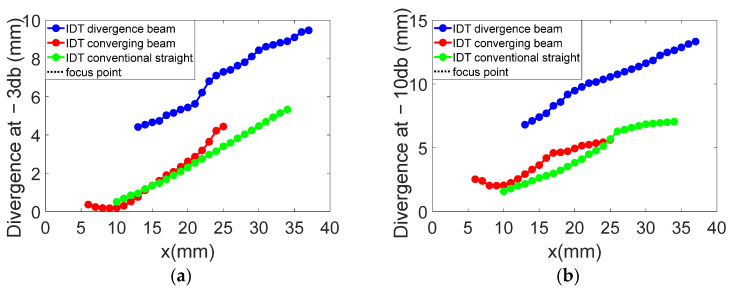
Comparison of beam widths in millimeters for the three IDTs: (**a**) at −3 dB; (**b**) at −10 dB.

For divergence angles, the results shown in Figure 25 indicate that all three IDTs produce divergent SAWs with respective angles of 14.7°, 15.2°, and 14.7° at −10 dB. The beam slopes are similar across the configurations, except for the convergent IDT beyond the focal point.

**Figure 26:**
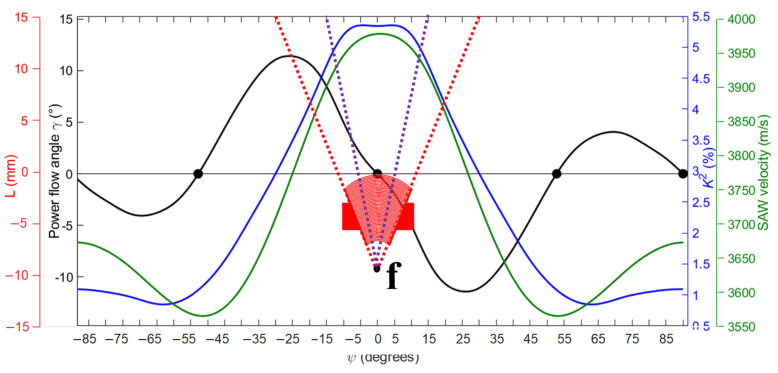
SAW propagation speed, coupling coefficient (K^2^), and power flow angle (ψ) as a function of propagation angle [31,32].

With regard to the divergence angles obtained on the three transducers, the results are shown in Figure 25 at −3 dB and −10 dB. The results show that all the solutions generate divergent SAWs, giving divergences of 14.7, 15.2, and 14.7°, respectively. The slopes of all the figures shown in Figure 26 are virtually identical. Of course, for the convergent IDT, only the part at the end of the focal point (x = 10 mm) should be considered.

From the results representing beam widths calculated from the displacement field profiles, an arctangent function was applied to determine the angles. The divergence angles for the three configurations are 14.7°, 15.2°, and 14.7° at −10 dB and 13.1°, 14.7°, and 11.6° at −3 dB. Although the designs of the three IDTs differ significantly, the divergence angles on lithium niobate are relatively close. For example, the divergent IDT was expected to produce a divergence of 60° based on its aperture, but the actual divergence remains limited to 14.7° at −10 dB.

These findings can be explained by the intrinsic properties of lithium niobate rather than the electrode designs. Weser et al. [30] presented the orientation-dependent properties of Rayleigh waves propagating on a LiNbO_3_ Y+128° crystal, in particular the coupling coefficient K^2^, surface acoustic wave (SAW) phase velocity *v*_p_, and power flow angle γ (Figure 26). The abscissa variable ψ is the propagation angle with respect to the X-axis of the crystal. These classical curves are based on numerical data found by Kovacs et al. [31] and used here to interpret our observations.

First, the divergence is limited by the anisotropic nature of the electromechanical coupling coefficient (K^2^) on Y128°-cut lithium niobate. For this crystal cut, the coupling coefficient (K^2^) varies with the SAW propagation direction. In the direction perpendicular to the flat edge (0°), the coupling coefficient is maximal at about 5.4%. As the propagation angle ψ changes, K^2^ decreases and becomes less than 1% at 60°.

Secondly, the divergence is also limited by the phenomenon of beam steering [32,33]. On an anisotropic substrate, the SAW does not always propagate in the initial direction. Due to the material’s properties, the wave energy can deviate from the wave vector direction. This deviation is characterized by the power flow angle γ, which represents the angle between the group velocity (actual energy propagation direction) and the phase velocity (wave vector) [29,33,34]. For Y128°-cut lithium niobate, γ is zero only at 0°, ±52.8°, and ±90°. Otherwise, beam deviation occurs [23]. For propagation angles within ±30°, the energy propagation direction is modified. For example, for a propagation angle of +20°, the energy direction angle is +10° (reduced by 10°). These two phenomena explain why the divergence is around 15° on Y128°-cut lithium niobate.

Moreover, the position of the focal point depends directly on the angle of propagation of the energy. The best focusing performance is obtained when the orientation-dependent group velocity converges towards the focal point from each section of the interdigital transducer (IDT) [35,36]. If this condition is not met, the focal point is shifted and spread over some regions (Figure 17).

## 8. Conclusions

In conclusion, this study demonstrated the possibility of obtaining the divergence of surface acoustic waves from IDT sensors. The three configurations considered achieved a divergence of around 15°. It was shown that the divergence achievable on lithium niobate was limited by the nature of the material, and that divergence angles greater than 15° were not possible due to the anisotropic coupling coefficient and beam steering. Finally, the aim of this study was to limit as far as possible the contact zone between the IDT and the sample in which the SAW reverberation is desired. The three configurations achieved contact widths of 10 mm, 3.4 mm, and 1 mm, respectively. The third configuration (narrow IDT) was particularly successful in significantly reducing the contact area. However, the second solution provides higher SAW amplitude, which is important while measuring the diffuse acoustic field.

## Figures and Tables

**Table 1:** Main parameters of the three SAW devices.

IDT	Number of Electrode Pair	Angular Aperture (°)	Curvature Radius (mm)	Length (mm)	Frequency (MHz)	Contact Area (mm^2^)
Divergent IDT	25	±30	2.8	7	15	10 × 1
Convergent IDT	25	±30	2.8	7	15	3.4 × 1
Narrow IDT	25	0	∞	6.6	15	1 × 1

## Data Availability

The data supporting this study are available upon request from the corresponding author.
